# Multiple myeloma presenting as acute tubulointerstitial nephritis

**DOI:** 10.4322/acr.2021.328

**Published:** 2021-09-23

**Authors:** Ying Long, Abed A. Aljamal, Hisham F. Bahmad, Niharika Yedla, Guillermo A. Herrera, Michael A. Schwartz, Ayman Layka

**Affiliations:** 1 Mount Sinai Medical Center, Department of Internal Medicine, Miami Beach, FL, USA; 2 Mount Sinai Medical Center, Arkadi M. Rywlin M.D. Department of Pathology and Laboratory Medicine, Miami Beach, FL, USA; 3 Louisiana State University Health, Department of Pathology Shreveport, LA, USA; 4 Mount Sinai Medical Center, Department of Internal Medicine, Division of Hematology and Oncology, Miami Beach, FL, USA; 5 Mount Sinai Medical Center, Department of Internal Medicine, Division of Nephrology, Miami Beach, FL, USA

**Keywords:** Case Reports, Multiple Myeloma, Nephritis, Interstitial, Microscopy, Electron

## Abstract

**Background:**

Acute tubulointerstitial nephritis (ATIN) is a very rare paraneoplastic manifestation in patients with multiple myeloma (MM). It is an uncommon pattern of renal disease in such patients.

**Case presentation:**

We report a case of an 82-year-old male who was admitted with acute kidney injury. Renal biopsy showed typical findings of light chain-associated ATIN with scattered inflammatory cells in the interstitium and associated active tubulitis. No other common manifestations of MM were present at the time of presentation, including hypercalcemia, hyperuricemia, proteinuria, bone pain or lytic bone lesions. Subsequent immunoassays revealed significant serum lambda light chain burden and Bence Jones protein in urine. Immunofluorescence demonstrated linear tubular basement membranes with positive staining for lambda light chain (3+). Electron microscopy (EM) further showed interstitial edema and inflammation. All the aforementioned findings are consistent with ATIN and supported the diagnosis of MM.

**Conclusions:**

In conclusion, light chain-associated ATIN should be considered in the differential diagnosis of acute interstitial nephritis. Henceforth, serum free light chains as well as serum and urine protein electrophoresis should be included in the workup of such patients.

## INTRODUCTION

Multiple myeloma (MM) is a plasma cell dyscrasia of terminally differentiated plasma cells.^1^ It is characterized by infiltration of the bone marrow with clonal plasma cells which secrete a monoclonal paraprotein or an immunoglobulin free light chain (FLC).^1^ It is the second most common hematologic malignancy accounting for 1–2% of all human malignancies.^2^^,^ 3 Renal failure, anemia, recurrent infections, and bone lesions are among the most common presentations of MM.^4^ Indeed, kidney disease is one of the major complications of MM and is found in 20-50% of patients at the time of diagnosis.^4^^,^ 5

Myeloma nephropathy occurs when the absorptive capacity of renal tubules for monoclonal immunoglobulin FLCs is exceeded causing casts to form within the tubules obstructing them.^6^ Not only FLCs can cause intratubular cast formation, but also isolated proximal tubule cytotoxicity, acute tubulointerstitial nephritis (ATIN), and/or light chain deposition disease.^7^ After ruling out reversible factors such as hypercalcemia, hyperuricemia, or nephrotoxic agents, the most common cause of acute kidney injury (AKI) in patients with MM remains tubulointerstitial pathology resulting from monoclonal immunoglobulin FLCs.^7^ Studies reveal that survival of MM patients is reduced to less than 1 year in cases of AKI if renal function is not recovered.^8^^,^ 9 In one study, for instance, the median survival was found to be 10.2 months among MM patients with severe AKI making early recognition a crucial prognostic factor.^8^

The principle tubulointerstitial lesions in MM include free light chain cast nephropathy, proximal tubulopathy without cytoplasmic inclusions (also referred to as acute tubular necrosis variant), and proximal tubulopathy associated with interstitial inflammatory reaction, which has pathological features identical to those seen in ATIN due to other etiologies.^7^^,^ 10 ^,^ 11 Free light chain cast nephropathy is the most frequent of those lesions and is well reported in literature.^12^ Although the pathological process could be recognized by a pathologist on a renal biopsy, the association of these lesions with the underlying plasma cell dyscrasia is often missed.

Herein, we report a case of an 82-year-old man who presented to our institution with acute kidney injury showing typical findings of light chain-associated ATIN on renal biopsy and supporting a diagnosis of MM. We further delineated the different pathological and electron microscopic features of this entity. This case report was conducted and reported in accordance with CAse REports (CARE) guidelines for reporting case reports.

## CASE REPORT

An 82-year-old man was brought to our emergency department after he was found lying unresponsive on the floor of his house. At the hospital, the patient reported having decreased oral intake, nausea, malaise, abdominal discomfort and decreased urinary output. His medical history was significant for hypertension, type II diabetes mellitus, abdominal aortic aneurysm, hyperlipidemia, congestive heart failure, deep venous thrombosis, pulmonary embolism, and chronic back pain. His medications included ibuprofen, oxycodone, gabapentin, atorvastatin, losartan, carvedilol, furosemide, ticagrelor, apixaban, pioglitazone, insulin glargine, sitagliptin, tamsulosin and escitalopram. He had no known allergies.

On examination, vitals revealed a temperature of 36.8^o^C, blood pressure of 113/68 mmHg, pulse of 65 beats/minute and a respiratory rate of 18 breaths/minute. Physical examination was unremarkable except for bilateral lower extremity pitting edema. Laboratory findings included a hemoglobin of 11.6 g/dL (reference range [RR] 14 - 18 g/dL), serum creatinine of 11.6 mg/dL (RR; 0.7 - 1.3 mg/dL), blood urea nitrogen (BUN) of 119 mg/dL (RR; 7 - 18 mg/dL), serum potassium of 6.0 mMol/L (RR; 3.5 - 5.1 mMol/L), estimated glomerular filtration rate (EGFR) of 4 mL/min/1.73m^2^ (RR > OR = 60 mL/min/1.73m^2^), serum phosphorus of 3.9 mg/dL (RR; 2.5 – 4.9 mg/dL), serum calcium at 9.2 mg/dL (RR; 8.6 - 10.3 mg/dL), albumin at 3.7 g/dL (RR; 3.4 – 5 g/dL), serum uric acid at 10.5 mg/dL (RR; 2.6 – 6 mg/dL) and a serum creatinine phosphokinase (CPK) at 327 U/L (RR; 26 – 192 U/L). Two months earlier, his serum creatinine was at 1.06 mg/dL, BUN at 14 mg/dL and EGFR>60 ml/min/1.73m^2^. Spot urinalysis revealed clear light-yellow urine with a specific gravity of 1.010 without any protein. The urine did not show significant white blood cells, red blood cells and was also negative for nitrites and leukocyte esterase. No urine casts were identified, and no eosinophils were noted. A catheter was inserted and urine sample from the catheter showed a spot urine microalbumin to creatinine ratio at 287.6 mg/g. Serum complement C3 and C4 levels were normal. Renal ultrasound demonstrated increased echogenicity throughout the kidneys, likely related to intrinsic medical renal disease without any hydronephrosis or nephrolithiasis.

**Figure 1:**
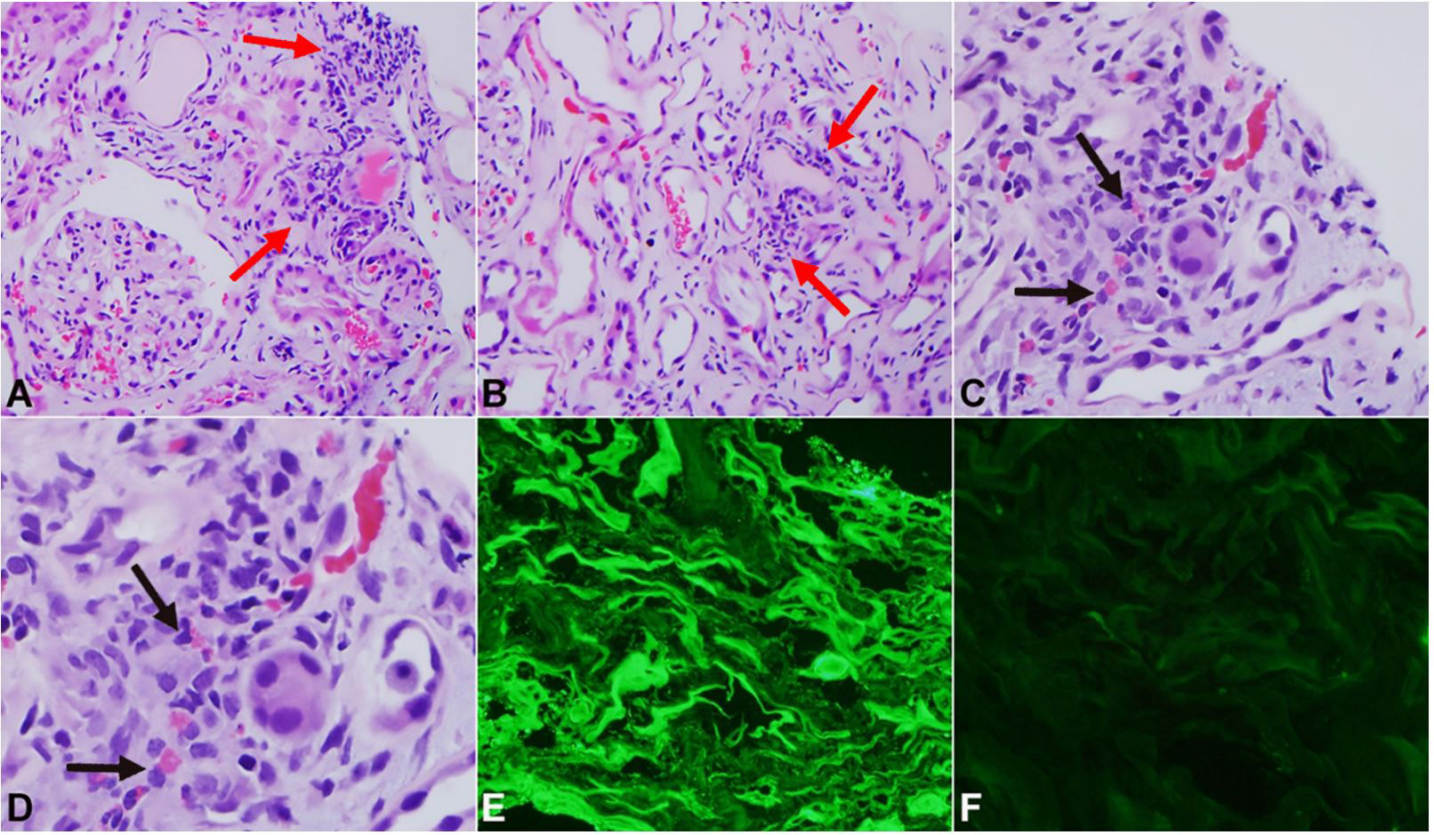
Microscopic and Immunofluorescence findings. (**A**) Interstitial inflammatory infiltrate (combination of eosinophils and mononuclear cells; black arrows) associated with tubulitis (red arrows), tubular damage, and interstitial edema. H&E x250. (**B**) Extensive tubular damage with simplification of tubules lined by flattened cells and interstitial inflammation with tubulitis. H&E x250. (**C**) and (**D**) Interstitial inflammation with tubulitis, edema, and tubular damage with eosinophils. H&E x250 in (**C**) and x500 in (**D**). (**E**) Immunofluorescence stain for lambda light chains, fluorescein isothiocyanate (x250) showing intense linear staining along tubular basement membranes and granular staining in the cytoplasm of some proximal tubular cells. (**F**) Immunofluorescence stain for kappa light chains (x250) with fluorescein isothiocyanate showing no staining in renal parenchyma.

**Figure 2:**
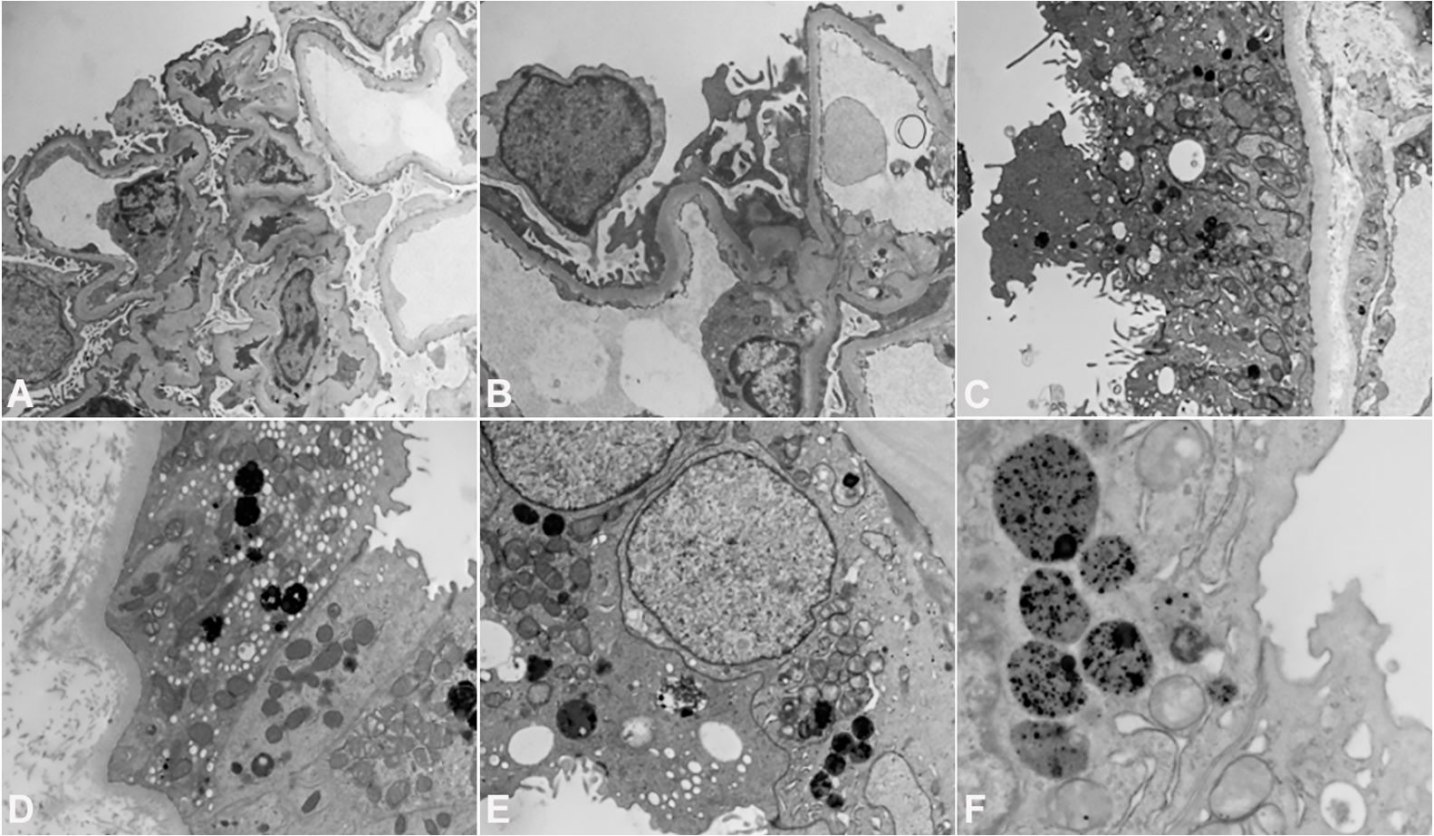
Electron Microscopy (EM) findings. (**A**) EM x7500 and (**B**) EM x12500 - Uranyl acetate and lad citrate stain showing no immune complexes, light chain deposits or fibrillary material. Mild, segmental effacement of the foot processes of visceral epithelial cells. (**C**) EM x7500 and (**D**) EM x7500 - Uranyl acetate and lead citrate stain showing apical blebbing, segmental loss of microvillous borders and lysosomes in cytoplasm of proximal tubular cells. (**E**) EM x12500 and (**F**) EM x18500 - Uranyl acetate and lead citrate stain showing details of lysosomes in proximal tubular cells. Note moth eaten appearance of lysosomes reflecting partial extrusion of their enzymatic contents.

On the third day of the admission, a renal biopsy was performed which showed global sclerosis in 2/9 examined glomeruli without any significant changes in the mesangium. The glomerular basement membrane was unremarkable. The main findings were in the tubulointerstitial compartment which showed evidence of tubular damage and tubulitis (Figure 1A-1D). The tubules appeared simplified and lined by flattened cells. There were scattered inflammatory cells in the interstitium which were a combination of eosinophils and mononuclear cells and were associated with active tubulitis. Some of the tubules appeared to be dilated and contained hyaline casts but no myelomatous casts were seen. There was a moderate degree of thickening of the walls of the arterioles along with the small and medium sized arteries. Immunofluorescence demonstrated linear tubular basement membranes with positive staining for lambda light chain (3+). In contrast, kappa light chain stain was essentially negative (Figure 1E and 1F). Electron microscopy (EM) revealed interstitial edema and inflammation without any immune complexes, fibrillary material, or light chain deposits in the tubulointerstitial compartment (Figure 2). No proliferation of lysosomes or atypical lysosomes were identified in the damaged proximal tubules. Increased numbers of lysosomes, a few atypical, were seen in selected proximal tubules. These findings were found to be consistent with ATIN. This raised the suspicion for a monoclonal lambda light chain-related tubulointerstitial process.

**Figure 3:**
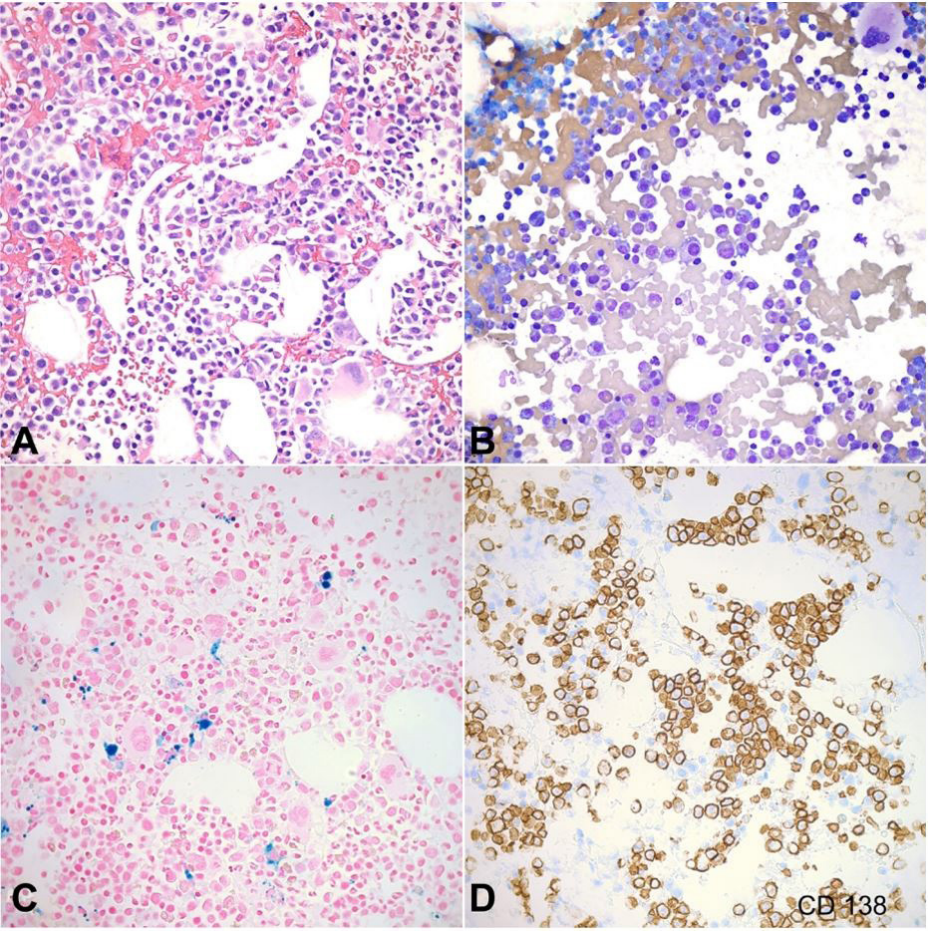
Bone marrow biopsy and aspirate findings. **A** – Bone marrow biopsy, and **B** – aspirate analysis showed a plasma cell neoplasm with lambda light chain restriction (H&E x400); **C** – Prussian blue staining revealed adequate iron storage (x400); **D** – CD138 stain was positive in increased numbers of plasma cells representing approximately 50% of the marrow cellularity (x400).

The findings on the biopsy prompted us to pursue the workup for multiple myeloma. We obtained a plasma protein electrophoresis which showed a peak in the beta-1 globulin zone and a monoclonal lambda protein was identified by immunofixation. Serum free light chains showed an elevated lambda up to 9503 mg/L and an almost normal kappa of 24.53 mg/L. Bence Jones protein was present in the urine. Given the significant lambda light chain burden, a diagnosis of monoclonal lambda light chain-related ATIN was made. Bone marrow biopsy and aspirate showed a plasma cell neoplasm with lambda light chain restriction (Figure 3A and 3B). Prussian blue stains of the biopsy revealed adequate iron storage (Figure 3C). CD138 was positive in increased numbers of plasma cells representing approximately 50% of the marrow cellularity (Figure 3D). Fluorescence in situ hybridization (FISH) analysis was also performed using a set of probes specific to multiple myeloma. Plasma cell enrichment was performed revealing various abnormalities including: 13q14 deletion (1R2G, 66.0%, normal < 11.9%), an atypical IgH gene rearrangement/partial gene deletion (1R, 64.8%, not seen in validation studies), and a gain in chromosome 1q (CKS1B) (3R2G 67.0%, normal < 8.6%). Counts for the remaining probes were within the normal reference range. No lytic lesions were seen on the bone survey. Laboratory tests were repeated showing a white blood cell count of 12.4 x 10^3^ /μL (RR; 4.8 - 10.8 x 10^3^/μL), red blood cell count of 2.7 x 10^6^ /μL (RR; 4.63 - 6.08 x 10^3^/μL), hemoglobin of 8.6 g/dL (RR; 14 - 18 g/dL), hematocrit 25.6% (RR; 42 - 52%), MCV 91.8 fL (RR; 79.0 - 92.2 fL), RDW 13.7% (RR; 11.5 - 15.0%), and platelets 182 x 10^3^ /μL (RR; 150 - 450 x 10^3^/μL). The diagnosis of multiple myeloma was hence confirmed.

**Figure 4:**
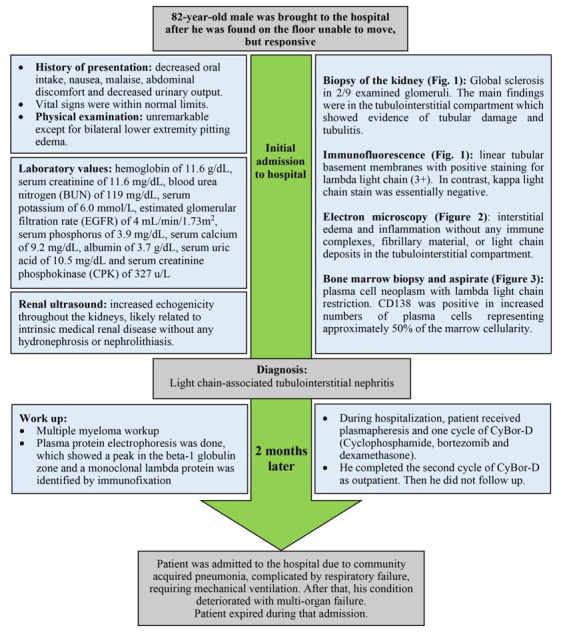
Timeline organizing main events of the case.

During hospitalization, the patient received plasmapheresis and one cycle of CyBor-D (Cyclophosphamide, bortezomib and dexamethasone). At the time of discharge (day 20 of the admission) serum creatinine was at 6.2 mg/dL, BUN at 100 mg/dL and the EGFR at 9 ml/min/1.73m^2^. One week after discharge, lambda free light chain was noted to be 3107 mg/dL. The patient completed his second cycle of CyBorD as an outpatient but failed to return for scheduled follow-ups. He was readmitted to our hospital 2 months later for a community acquired pneumonia complicated with respiratory failure requiring intubation and pressor support. During this hospitalization, his mentation worsened; he had episodes of cardiac arrhythmias with worsening kidney function. The patient’s family decided to transfer him to hospice. At the time of writing our case report, the patient had already died (Figure 4).

## DISCUSSION

Renal diseases associated with multiple myeloma (MM) are usually due to the deposition of monoclonal light chains within the renal parenchyma. Some light chains interact with Tamm-Horsfall protein in distal tubules forming casts and disrupting tubular basement membranes which eventually causes acute tubular interstitial nephritis (ATIN) with multinucleated giant cells.^10^ Light chain-associated ATIN is characterized by interstitial inflammation and tubulitis. A prominent finding at areas of inflammation is the presence of linear – sometimes interrupted – monotypical light chain staining along the tubular basement membranes. In some cases, there is also monotypical light chain staining in the cytoplasm of proximal tubular cell. The tubules also typically contain an abundance of lysosomes, some atypical with evidence of tubular damage, with associated areas of inflammation and tubulitis. Deposition of punctate electron-dense material along the tubular basement membrane is noted in some cases but is usually focal and unimpressive.^11^ The mechanism underlying ATIN by light chains remains speculative with hypotheses pointing to a role that cytokines might be playing in this context,^10^^,^ 13 inducing chemoattraction and activation of interstitial mononuclear inflammatory cells.

Renal biopsy in our case of light chain-associated ATIN showed the typical findings of the disease: an inflammatory tubulointerstitial process with linear staining for lambda light chain (3+) along the tubular basement membranes. It also revealed vascular nephrosclerosis and moderate glomerulosclerosis, both of which are consistent with the patient’s long-term history of hypertension and diabetes. In our case, acute kidney failure would be best explained by the ATIN process related to light chains. Although the use of non-steroidal anti-inflammatory drug (NSAID) by the patient could cause ATIN, the positive immunofluorescent stains for lambda light chains would not be expected in such cases, and the renal function in case of NSAID-induced ATIN generally recovers within weeks to a few months of treatment which was not the case with our patient.

AKI can be the first clinical presentation in patients with MM, as in our case. Yet, our patient did not have any of the other typical manifestations of MM such as hypercalcemia, hyperuricemia, proteinuria, bone pain or lytic bone lesion at the time of presentation. ATIN was the only clinical presentation of MM. Therefore, recognition of the association between tubulointerstitial nephritis and monoclonal light chains becomes extremely important for early diagnosis of MM. In a retrospective study by Herrera *et al*.,^11^ among all the renal lesions identified to be associated with monoclonal light or heavy chains, 22% were ATIN, as compared as 33% with light chain casts nephropathy, which is more commonly associated with MM. Tubulointerstitial nephritis, although an uncommon pattern of renal disease in a patient with monoclonal gammopathy including MGRS or MM, is not a rare presentation of monoclonal gammopathy.

The international myeloma working group considers only renal failure caused by light- chain cast nephropathy as a myeloma-defining event. Other forms of renal disorders related to MM, such as light chain amyloidosis, monoclonal immunoglobulin deposition disease, and light chain Fanconi syndrome, can occur independently without evidence of myeloma-defining events.^3^ These types of renal disorders caused by nephrotoxic monoclonal immunoglobulin are currently considered to be added under the term of monoclonal gammopathy of renal significance (MGRS).^14^

In conclusion, studies have shown a significant incidence of tubulointerstitial nephritis in patients with monoclonal gammopathies. Light chain-associated acute tubulointerstitial nephritis should be considered in the differential diagnosis of ATIN. Also, serum free light chains as well as serum and urine protein electrophoresis should be included in the workup of such patients. Since in our case, the patient did not have any other myeloma-defining events, questions rise about the association of ATIN and MM. Could ATIN be considered a characteristic of MM or a type of renal manifestation of MGRS? And more importantly, if light-chain related ATIN is observed in a patient without any myeloma-defining events, should chemotherapy be initiated? More studies need to be conducted to solve this dilemma and give definitive answers.
